# Collectanea: Pathology and Practice

**Published:** 1834-07-01

**Authors:** 


### COLLECTANEA.

### PATHOLOGY AND PRACTICE.

### ON THE CIRCULAR AND FLAP OPERATIONS.

There is a curious article in the American Journal for February, 1834, in which the relative merits of these methods of operating are estimated on geometrical principles. Dr. Tolefree, the author, offers some objections to a statement made by Sir George Ballingall, as to the relative quantities of cut surface exposed in the circular and flap operations.

Sir George, it seems, with the help of two mathematical friends, has come to the conclusion that these surfaces are nearly in the ratio of 7 to 15, and hence argues in favour of the circular method. His reasoning is founded on the following suppositions: that the areas exposed are respectively those of a circle perpendicular to the bone; and of an ellipse whose major and minor axes are as 2 to 1, which will be the case if the length of each flap be equal to the breadth of its base.

Dr. Tolefree, on the other hand, contends that this length of flap is greater than is recommended by the most violent flappists; and that even the warmest votaries of the circle admit the advantage of two incisions, one a little behind the other, instead of a single one, so as to give the stump the shape of an inverted cone; the superficies of which, as he justly argues, would be considerably greater than that of a mere circular section of the limb; in fact, if it were rectangular, even taking Sir George's account of the flap, the ratio of the surfaces would be 47 to 56, or about 4 to 5; and the argument in favour of the circular incision must of course be reduced in the same proportion.

It should however be observed, on the side of Sir G. Ballingall, that the *cut*, as distinguished from the *exposed* surface, would still be that of a circular section, an argument of some weight where the feelings of the patient are to be consulted; for, instead of a whole circle, it will now consist of two circular rings in parallel planes, one behind and within the other, down to the bone. Moreover, though we admit, with Dr. Tolefree, that "the reader, in this computation, must not forget that those who calculate in the manner of Sir George Ballingall base their reasoning on the supposition that the stump of the bone is cut in a pyramidal shape, when, the truth is, it is the same as in the circular operation;" (p. 373,) we suspect that he has forgotten, on his own part, a certain proposition in the fifth book of Euclid, about the effect of taking equal parts from quantities which bear to each other a ratio of less inequality. He also seems to have forgotten that the contraction which takes place after the cut must draw the base of the aforementioned cone into a smaller circle, and thus modify the computation of its surface once more in favour of Dr. Ballingall, considerations which so minute a critic should not have failed to notice.

Our own opinion is, that the contraction of the soft parts, including the skin, practically does away, in a great measure, with any supposed advantage of the flap operation. We once saw this strikingly illustrated after an amputation of the humerus: the skin, partly by its own contractility, and partly by its adhesion to the edge of the retiring muscle, was drawn smoothly in, all round, so much as almost entirely to cover the face of the stump before the dressing was applied: and the same thing takes place in amputations of the thigh, though not perhaps to the same extent, or so soon as in the case we have mentioned; particularly when the limb is removed soon after an injury, while the skin is healthy, and, with the other integuments, retains its natural state of tension.

The advantages of this operation over the flap are, that it is shorter and less painful; that it saves a thicker mass of flesh to cover the end of the bone; that it exposes, at any rate, a less extent of cut surface; and, above all, that it requires a very much less extensive division of the skin; a point, which, though not adverted to by either party, (at least, if we may judge of Dr. Ballingall by Dr. Tolefree's extracts,) we do not think it necessary to prove by a tedious rectification of the ellipse formed by the periphery of the double flap.

We cannot refrain from adding our belief, that the well-doing of a stump depends much more upon light and careful dressing, and upon efficient attention to the health of the patient both before and after the amputation, than upon the choice exercised as to the particular mode. For proofs of this we would appeal to the comparative result of cases managed at our large hospitals by any attentive dresser during the first and last part of his period of attendance; and to the mortality and deformity noticed in cases of this kind at the Paris hospitals, as contrasted with the general success observed in our own.

### CONDYLOMATA CURED BY CREOSOTE.

Dr. Fricke, director of the surgical division of the Hamburgh Hospital, has tried the external use of creosote with great success, in the most obstinate forms of condylomata. The pointed ones, which are the most difficult to remove, were touched with a hair-pencil dipped in diluted creosote. This whitened the surrounding parts, but only temporarily, as the whiteness generally disappeared within half an hour. The condylomata themselves did not change colour till after the lapse of a couple of hours, when they became white, or sometimes brownish. In twenty-four hours a part of the condyloma had died off, and lay upon the remaining portion, or hung from it. The cuticle also died off, but was immediately replaced by a new one, so that there was nothing like excoriation to be seen. Small condylomata disappeared after one or two applications, but large ones required more. Some few very obstinate cases were not cured in less than a fortnight or three weeks: but once removed, the condylomata never returned.

Should the efficacy of creosote in this disease be confirmed by other observers, it would be an inestimable remedy, as it removes the excrescences without excoriating the neighbouring skin. This cannot be said of any other remedy hitherto known. Those which we consider drying applications, such as the Extr. Saturn. (Goulard's wash,) act in a totally different manner. Dr. Fricke has not seen any particular effect produced by creosote when applied to ulcers, cancers, fungus hæmatodes, &c.—*Abridged from the Wochenschrift für die gesammte Heilkunde.*

### IN WHAT DOSE SHOULD DIGITALIS BE GIVEN?

We had thought that this question was long ago settled, and that those who ventured on the administration of this tremendous medicine were content with doses of half-a-grain or a grain three times a day. It would appear, however, from two papers, by M. Joret, in the *Archives Génerales* for January and March, that, in the Hospitals of Paris, it is a common thing to give a patient ten or fifteeen grains of powdered digitalis in a day; and in one case the dose was carried to forty-eight grains! In another series of cases the aqueous extract was given, in another the alcoholic extract, and in another the ethereal extract. In the fifth series of cases the digitalis was used in infusion, administered by the mouth and per anum. In one instance the infusion of three ounces and twelve grains of the fresh leaves was taken in eight days. These enormous doses frequently excited colic or diarrhœa, and still more frequently the most violent vomiting. No one of the patients, however, died. The author adds three cases, treated by M. Gendrin, at the Hôtel Dieu, in two of which the termination was not so fortunate. They will be a valuable addition to the next edition of Dr. Christison's work.

Case I. A man, about forty-five or fifty years old, strong and robust, was admitted into the Hôtel Dieu, with a quick pulse, and all the symptoms of hypertrophy and dilatation of the heart. M. Gendrin prescribed, on the first day, a potion made with an infusion of half a drachm of the dried leaves of digitalis. The next day the patient said that he was better, the pulse was slower, and he had suffered from nausea in the night, but had not vomited. M. Gendrin, supposing that the patient was about to become accustomed to the medicine, prescribed a drachm of the dried leaves in an eight-ounce potion. On the following day the patient had still experienced nothing but nausea: his pulse was slow. Half a drachm of the dried leaves was prescribed. The patient, as well as I recollect, says M. Joret, only drank half his potion, and saying that he was cured, refused to take the rest. Three or four days passed away, and he was well enough to walk about and play at cards with the other convalescents. At the end of this interval, the patient, on coming out of the necessary, fell like a log upon the pavement; the nurses ran to help him up, but he was dead.

CASES II and III. M. Gendrin began by giving an infusion of four drachms of the dried leaves to each of two patients in the St. Landry ward: one was consumptive, and the other had an eccentric hypertrophy of the heart. The first vomited twelve times in the twenty-four hours; the second only seven or eight times. Nevertheless, the physician of the Hôtel Dieu, always supposing that they would become used to the medicine, prescribed an infusion of five drachms to each patient.

The consumptive patient offered to give up his ticket, preferring, as he said, to leave the hospital, rather than vomit again. The other one was more courageous, and drank the whole of the prescribed potion. Although the dose of digitalis was greater, the patient vomited much less than on the first day; the pulse, which, before the administration of the digitalis, was 120, had sunk to forty-five. The patient was exceedingly contented, and said that he felt quite comfortable. The potion was omitted, and two days had passed away, when, on seeing out his relations, who had come to visit him, he fell on the edge of the staircase, as if he had been struck with lightning, and was carried to bed dead.

Nothing was found on dissection to explain the cause of so sudden a death. The heart was hypertrophied to the highest degree, but there was neither laceration, nor any other lesion. The right ventricle contained a large clot of black blood, which filled its cavity; and similar clots were found in the large venous trunks. The vessels of the stomach were merely injected, and had not the red tint which is found in acute gastritis. There was no morbid appearance in the brain, but a slight injection of the membranes.

Patients suffering from diseased heart were at that time very numerous in M. Gendrin's wards, as he was one of the physicians of the *bureau central*, and sent himself cases of this kind; but Dr. Joret never saw the patients get used to the medicine, though he saw several recovering perfectly after doses of digitalis carried as high as two drachms.

M. Joret is of opinion that the infusion is the most active of the preparations of digitalis, that the powder may be given in daily doses of twelve or eighteen grains, beginning however with one only; that the aqueous extract may be given in higher doses than the powder, without irritating the alimentary canal; and that the alcoholic extract, the ethereal extract, and the ethereal tincture cannot be trusted to.

### RUPTURE OF A VARICOSE TUMOUR IN THE VAGINA DURING LABOUR.

A woman, about thirty years old, whose previous pregnancies and labours had not been attended by anything extraordinary, observed, at the end of her third pregnancy, a soft tumour protruding from the vagina. The midwife was called in, and recommended a bleeding, which relieved the patient, but did not remove the disease.

The tumour continued to enlarge, but the woman was well in other respects, and never complained of pain. During labour, the very moment that the head entered the pelvis, the tumour broke, and discharged six or seven pounds of blood. The patient immediately fainted, her extremities became cold, and she remained devoid of consciousness. I was sent for instantly, (says Dr. Steudel, the narrator of the case,) and went to her, accompanied by an accoucheur. All our efforts to revive her were useless: the tumour was empty, and its cavity was big enough to contain one's fist. We endeavoured to apply the forceps; but, as it always slipped off, turning was practised. The child, when it came into the world, was dead, and had a spina bifida.—*Archives Génèrales, from the Medizinisches Correspondenz—Blatt.*

### SNEEZING.

The act of sneezing is produced by irritating the sensitive extremities of the branches of the fifth pair of nerves distributed to the pituitary membrane, which, by the connexion of that nerve with the eighth pair, the great sympathetic and the phrenic nerves, calls into simultaneous action the diaphragm and the whole of the respiratory muscles so suddenly, after a full inspiration, as to expel the air from the lungs forcibly through the mouth and nostrils. It tends to clear the nostrils of concreted mucus, and so far may be productive of benefit. Thus, in a case which lately came within my knowledge, a lady was afflicted with violent headach, accompanied with that sensation which is well known by the term stuffing of the head. Many means tried for her relief proved ineffectual. A physician was called in, who prescribed snuff as a sternutatory: it produced violent sneezing, and ejected from one of the nostrils a plug of hardened mucus, nearly an inch in length; after which she felt immediate relief, and in twenty-four hours was perfectly recovered. In some cases, however, sneezing is productive of the most serious mischief: for instance, a young lady, in whom there was an affection of the ethmoid bone, was attacked with sneezing, arising from some accidental circumstance: in a few hours it proved fatal. Many instances, also, are recorded in which sneezing has produced immoderate bleedings from the nose, epileptic fits, and apoplexy; and, consequently, those errhines which were formerly regarded as sternutatories are now seldom prescribed with the view of producing that effect of errhines.*—*Dr. A. T. Thomson's Elements of Materia Medica.*

* "A singular fact connected with this subject came under my observation. A lady, liable to periodic attacks of gout, was always apprised of the approach of the paroxysm by successive fits of sneezing, which generally continued for ten or twelve hours previous to the commencement of the attack, and terminated when the pain was felt in the foot. I can account for this circumstance only by supposing, that the gouty diathesis so altered the usual state of the pituitary membrane as to render it susceptible of the impressions even of the air; and thence it is possible that, although the mucous follicles are not excited to a degree sufficient to enable them to empty their contents, yet the passage of the air, in a highly irritable state of the membrane, may be sufficient to induce the paroxysm of sneezing, previous to the attacks of gout."

### CASE OF DISEASE OF THE SYMPATHETIC NERVE.

William Sharpe, eleven years of age, had a purulent discharge from the left ear, which began in the spring of 1824, kept gradually increasing, and became very offensive. Blisters were applied many times behind the ears, and tonic medicines administered without producing any good effect on the local disease, although his general health was improved by them. About the end of March, 1825, the integuments around the ear became swoln and painful, and the whole side of the face was enlarged. At this time the discharge was very copious and offensive; he had frequent pains in the head and over the left eye. On passing a probe into the meatus the bone was found to be denuded; his health had declined, and tonic medicines were of no use. In December it was observed that the left side of his face was nearly paralytic; he had violent pains in the head and face, which were much aggravated at night, and for which he took an opiate with some relief. He was frequently drowsy, and sometimes nearly comatose. In February, 1826, there was some inflammation of the conjunctiva of the left eye, which went off in a few days. On the 12th of October the left temporal bone appeared to be quite loose in the wound, and was easily extracted. About a week before this time his right eye became amaurotic, the pupil was dilated, and the lids closed. On the 14th he became insensible, but cried out when touched. On the 16th a large vesicle formed at the inferior part of the left cornea, and the greatest part of the cornea had become opaque. The right cornea was not altered. He had slight convulsions, and when the left side of his face was touched he flinched. He died in the night.

He walked about until the last few days. He had generally a good appetite. His food appeared to digest properly, and indeed all the functions of the viscera of the chest and abdomen were perfectly performed. He could talk distinctly. The left side of his face was nearly paralytic, and the left side of the nose was completely drawn to the right side. When he cried out, so as to exert the muscles of the face much, it was observed that those of the right side had very great power over those of the left; but the mouth, when shut, appeared even, and would not then have been supposed to be paralytic. From the end of last March the pain in the right side was very severe, he shrieked very much, and the opiate did not relieve him. His manner became altered, and he was unwilling to answer questions. He was generally easier in the day, and spent much of it sleeping in the sun.

*Examination.* This took place on the 17th of October, at eleven A.M. The dura mater was rather more vascular than is usual. The arachnoid membrane was much thickened, and especially on the right side, and there was much fluid between it and the pia mater. The pia mater was very vascular. There was much fluid in the lateral ventricles. The portion of brain which lay over the part from which the temporal bone was separated was protruded into a hernia, and the inferior cornu of the lateral ventricle was thereby drawn out of its course; the brain at this part was softer than natural, but the rest of it was sound. The origins of the nerves were distinct.

The third branch of the fifth pair was in a state of ulceration, near its beginning from the Gasserian ganglion; the gustatory, dental, and buccal nerves were however quite attached to it, and indeed di l not appear to have suffered.

The auditory and facial nerves terminated in a bulbous mass on the dura mater. The facial nerve could with difficulty be traced in the face near the edge of the jaw, on account of the inflammatory process which had been going on there. It could, however, be distinctly seen to communicate with the dental nerve, and then terminate in a confused mass, which formed the walls of the cavity containing the exfoliating bone.

The par vagum, the glosso-pharyngeal, and the accessory nerves were sound, and passed just behind the walls of the same cavity.

The internal carotid artery, from above and below, could be traced as far as the walls of the cavity, and was then lost. It was reduced to a small size, however, before it reached the walls of the cavity, and was impervious.

The superior cervical ganglion of the sympathetic nerve terminated in the walls of the cavity. The branches given off from the sixth, which usually go to the superior cervical ganglion, were very small, and terminated with the internal carotid; the superior cervical ganglion itself appeared natural.

The vidian nerve was perfect from its connexion with the spheno-palatine ganglion. The superior branch could be traced a short distance, but soon became much smaller than usual; the inferior branch could be traced a little way, it then became very small, and the branches from the sixth could be traced the same distance, and both of these terminated with the internal carotid artery, in the walls of the cavity.

The condyloid process of the lower jaw was exfoliating. The whole of the temporal bone had exfoliated, and to compensate for it, the orbital plate of the frontal bone had become unusually thick, and all the bone in the neighbourhood of the disease had a firmer texture than is observed at his age. The pericranium round the opening at which the hernia protruded, was much more vascular than in the other parts.

A considerable portion of the brain had protruded like a fungus into the cavity left by the exfoliated bone; it had been forming gradually, and was, no doubt, the cause of his death.

The quantity of fluid contained in each ventricle was the same, and therefore the blindness of the right eye did not depend on this as its cause, for vision remained in the left after the right one was blind.

In the examination, a black pin was found in the cavity, which had contained the exfoliated bone. Pins had not been used to confine the dressings, the question therefore is, whether it had been forcibly introduced into the ear, and occasioned the disease, but this could not be ascertained.

It will be seen, from this very curious case, that the sympathetic may be partly destroyed, and the general health remain unimpaired, as we have a right to presume after a due consideration of all the circumstances which have been related. We may conclude, therefore, that although the sympathetic nerve produces a general sympathy in the body, yet that each ganglion has a somewhat local influence, inasmuch as it more particularly connects the parts giving and receiving branches from it, so as to associate them in complicated operations. Both the spheno-palatine ganglion, and the vidian nerve, were first of their usual size, then each branch of the vidian was diminished. Can it be presumed from this, that the branches of the vidian are going to, and not coming from, the sympathetic nerve?

The functions of the facial nerve must have been entirely suspended, and, from the evenness of the mouth when at rest, and from his ability to speak so well, I cannot help concluding that the branches of the fifth had very considerable power in exciting the action of the muscular structure of the lips.—*Swan on Diseases of the Nerves.*

### TREATMENT OF THE VENEREAL DISEASE IN THE HOSPITAL OF THE 72D REGIMENT, AT THE CAPE OF GOOD HOPE.

| Years. | Primary Venereal Disease treated. With Mercury. | Primary Venereal Disease treated. Without Mercury. | Total. | Secondary Symptoms after Mercurial Treatment. | Secondary Symptoms after Non-Mercurial Treatment. | Relapses after Mercurial Treatment. | Relapses after Non-Mercurial Treatment. | Total. |
|---|---|---|---|---|---|---|---|---|
| 1829 | 39 | 57 | 69 | 4 | 0 | 1 | 0 | 5 |
| 1830 | 40 | 64 | 104 | 4 | 2 | 2 | 0 | 8 |
| 1831 | 25 | 71 | 96 | 3 | 6 | 3 | 1 | 13 |
| 1832 | 12 | 30 | 42 | 2 | 10 | 6 | 2 | 20 |
| Total | 116 | 222 | 338 | 13 | 18 | 12 | 3 | 46 |

*Mr. T. Clarke, in Med. Gazette.*

### DEEP-SEATED ABSCESS OF THE THIGH.

After the lecture, a patient was brought into the hospital under the following circumstances. He stated that he was a hackney car-driver, and aged about thirty-five years. Five days previously he was seized with severe pain in the back of the thigh, and on the following day he was unable to straighten his knee. Since the commencement of the pain he has felt " a great sickness over him," and has never enjoyed a single hour's sleep. He looks exceedingly ill; his pulse is small and frequent; there is a light erysipelatous blush over the integuments at the back of the thigh; the whole limb appears to be somewhat swelled; and Mr. Crampton pointed out to the class, that it was tense and hard at its posterior part, a little above the ham.

"This, gentlemen," said Mr. Crampton, " is a most important and instructive case. It is an instance of acute deep-seated abscess of the thigh, with its constant attendant, erysipelas of the skin. I shall give you a particular lecture on this case; but at this late hour all that I can do is, to show you what is required for the safety of the patient. The matter, which has just been formed, is lying deep beneath the fascia, and even below the superficial muscles. If art were not to interpose, the suppuration would extend upwards and downwards among the muscles, producing intense pain; and if the man were (as he appears to be) of a bad constitution, the inflammation would assume the erysipelatous character, in which, you know, the adhesive process is deficient, and spread all through the limb. The cellular membrane, deprived of its vitality, would form sloughs immersed in a sanious suppuration; the skin, detached from the superficial fascia, would become pale, then purplish, and this would be the precursor of its sloughing; eschars would form on it from its partial mortification, and when these separated (if the patient survived the mischief,) it would be found that the openings led into an immense cavity filled with unhealthy pus, and traversed by muscles altogether deprived of their cellular texture, and appearing more clean than you have ever seen them on a dissecting-table. Concurrently with all this terrible local mischief, we should have suppurative adynamic fever of the worst description, profuse perspirations, diarrhœa, and not unfrequently purulent depositions in the lungs and liver. I need scarcely say that few indeed have a constitution capable of holding out under such accumulated sufferings. Accordingly, phlegmonoid erysipelas of this kind is not only one of the most painful, but one of the most fatal occurrences which we ever encounter; and here we have an illustration of the truth of the aphorism of Hippocrates respecting erysipelas: " Ex erysipelate putrido, aut suppuratione malum." You will observe, that the word " putrido" is used to express the sloughing and putrefactive process which characterizes the suppuration of erysipelas, as distinguished from that of phlegmon. Children escape oftener than adults; but I have very seldom indeed seen a man after fifty who has survived phlegmonoid erysipelas of a lower extremity, where the disease had got extensively into the intermuscular cellular membrane. The successful management of this disease I look upon to be the greatest triumph of modern surgery. You all know how much stress I have ever laid on the necessity of early and vigorous measures in the treatment of this affection; and I trust that the present case will add one more to the many successful cases which we have had of this disease since our session has commenced. Among others, the boy in the large clinical ward, with phlegmonoid erysipelas of the knee and thigh from a wound; the man with a similar affection of the fore-arm, from a wound on the elbow; the woman with erysipelas of the fore-arm and arm, from a slight contused wound of the elbow, on whom I operated last week, and whom you saw this morning among the externes nearly well, are all cases of the successful treatment of this disease.

It affords me great satisfaction in having an opportunity of rendering that justice to Mr. Copland Hutchinson which some of his own countrymen have denied to him. To him we are exclusively indebted for this great improvement in the treatment of erysipelas. Like all great improvements, it was at first met by a denial of its utility, and then by attributing it to somebody else; but I have no hesitation in thus publicly offering my acknowledgments to him for one of the most valuable improvements which have been made in the practice of surgery within our times.

The case before us, you will observe, is not one of phlegmonoid erysipelas, properly so called: it is a case of deep-seated intermuscular phlegmon, accompanied with erysipelas of the skin and subjacent cellular tissue, in consequence of the inflamed and tense state of the fascia. But in a constitution like this man's, broken down as it is by intemperance, the true phlegmonoid erysipelas would in all probability arise, if the abscess were not opened, and the tension of the inflamed fascia relieved.

Mr. Crampton having laid the man on the table in a prone position, made an incision six inches long over the posterior aspect of the lower third of the thigh. The cellular substance, which was now exposed, was loaded with a reddish coloured serosity. Mr. C. then slit up the fascia to the same extent, and laying aside the knife, he slowly insinuated his fore-finger between the muscles, just above the popliteal space, until he reached the cavity of the abscess, and this did not occur until his finger was buried as deep as it could go.

"Now," said Mr. Crampton, "I have reached the matter;" and, on withdrawing his finger, about eight ounces of thick cream-coloured pus gushed from the wound. A thin dossil of lint was interposed between the lips of the wound, and the man was carried to bed. He was ordered some purgative pills, and an effervescing mixture made in an infusion of cinchona.

Dec. 2d. The man is free from all complaint, and the wound is healing rapidly.—*London Med. and Surg. Journal.*

### ON THE DEATH OF NEW-BORN INFANTS DEPENDING ON ANORMAL STATES OF THE UMBILICAL CORD. BY DR. KOHLSCHWETTER.

The funis may cause the death of the child, 1st. by its prolapsus; 2dly, by becoming knotted; 3dly, by being twisted round the child; 4thly, by its shortness; 5thly, by the situation in which it is placed in a footling delivery. Authors do not agree in the cause of death in these cases; some attribute it to anemia, others to apoplexy, others to asphyxia, or rather the loss of the pulmonary function; and a fourth class, to the deficiency of nutritive juices which the funis can no longer transmit to the fœtus. Among these opinions we shall notice those alone which have enjoyed some degree of reputation.

1st. *Anemia.* Minelhauser advised (in 1754) that the funis should be cut and tied to prevent the child dying of anemia, in cases of prolapsus of the funis, or during a footling labour. This opinion was adopted by Stolper in 1807; and, according to this author, the tunics of the umbilical arteries are thicker and have more elasticity than the vein, and the blood circulates with more force in the former vessels. Hence the arteries will be less exposed to compression than the vein, and the blood not being returned, anemia will arise from hemorrhage. He thinks that the truth of this theory is demonstrated by the paleness of the child, and by internal signs of hemorrhage; but his opinion has not been corroborated by dissections. This theory is obviously improbable to the last degree, for it is not only opposed to the structure of the funis, which does not allow the vein to be compressed without the arteries, but moreover, in children who have sunk under pressure of the brain, there has never been a deficiency of blood, which is the only sign capable of demonstrating that hemorrhage was the cause of death.

*Plethora.* The authors who suppose that death takes place from this cause, by no means agree as to the kind of plethora which arises, for, according to some, it is general, and, according to others, partial. The reasons they urge in favour of their opinions are diametrically opposed to the writers before mentioned; for those with whom we have to do at present suppose that it is not the vein but the arteries that are compressed. It should not be forgotten, however, that there is no mechanical obstruction to the circulation, even if the umbilical vessels are obstructed. And, in fact, the pulsations of the heart often continue for more than ten minutes in children born in a state of asphyxia, from compression of the cord.

*Medico-legal Questions.* The different conditions of the funis may lay the foundation for so many difficult questions in medical jurisprudence. First of all, we must not forget to declare that respiration, while the child is in the vagina, is a possible occurrence. Artificial insufflation of the lungs may give rise to great difficulties; for if it is true that a congestion of blood may take place in the lungs of the fœtus which has not breathed, in consequence of the suppression of the umbilical circulation, we lose the diagnostic sign by which it has been attempted to ascertain whether the respiration has been natural or artificial. This is one of the weightiest objections brought forward by Jaeger against Plouquet's test.

Knebel and Bernt have supposed that prolapsus of the funis may be known to have taken place by the contusion and ecchymosis of the parts of the cord which have been compressed; and in a case of prolapsus Michaelis observed two red and ecchymosed spots on the cord. But this sign is not always present, for the pressure on the cord is often not great enough to cause ecchymosis. The attention of the physician ought to be particularly directed to the head, where he will find the traces of a bloody tumour, even if it has already disappeared; for if the child dies during the beginning of labour, the swelling is then to be seen in the middle of the sagital suture, whereas in new-born infants it is in the upper and posterior quarter of either parietal bone. Moreover, it must be remembered that children may be born alive, in spite of the prolapsus of the funis, but in such a state as to die after having breathed feebly.

When a knot upon the cord has been produced a long time before delivery, it leaves furrows in the spot where it has existed, as a trace of the meeting of the parts which had formed it. The very moment that the knot is untied, the cord swells a little, and shows such a tendency to remain knotted, that, if it is relaxed and pulled out, it immediately returns to its former state. But it is not so with a knot of recent formation; when once undone it leaves no trace behind.

The twisting of the cord around the neck of the child is a source of much doubt to the medical jurist; for neither experiments on the lungs, nor the ecchymosis around the neck of the child, nor the external and internal signs of asphyxia, will empower him to decide whether the death of the child has been produced by accident or design. A very remarkable case is mentioned by Jaeger. A woman was secretly delivered of a child, who came into the world in a state of half-asphyxia, the cord being twice twisted round its neck; and the mother was accused of having strangled it. There was a deeper and narrower mark around the neck of the child than the cord would have made; there was no ecchymosis, and the lungs were but little expanded by air. The cranial and thoracic cavities contained a great deal of blood. The question was ably resolved by the College of Physicians at Wurtemberg, and their decision was confirmed by the confession of the prisoner. In Servaès' case, the mother strangled the child with the funis itself. The fact narrated by Schwarz is still more replete with difficulties. In a face presentation, the child came into the world dead, and with its neck tightly squeezed between two circumvolutions of the funis. A livid zone surrounded the neck, causing a depression sensible both to the sight and touch. The face was rather swelled, but not livid, and all the rest of the body was covered with livid spots. The coronary vessels of the heart and stomach were gorged with blood, as well as the right auricle; the left auricle was empty and flabby. The lungs seemed gorged with blood, and sank in water. The veins of the abdomen were swelled; there was no congestion in the brain. If to this we add, that the child, presenting by the face and in the most favourable position for respiration in the vagina, might have breathed, we shall have all the signs of suffocation by strangulation. It is therefore with justice that Plouquet, Knebel, Henke, and others, have considered the solution of the problem in similar cases as exceedingly difficult. According to Adolphus, Plouquet, Henke, Hinze, Platner, and Bernt, the twisted cord may leave a livid zone around the child's neck. Klein and Elsasser are of the contrary opinion, and do not allow the possibility of a zone produced by the funis during labour. Albert observed a livid mark situated in the axilla, seven lines long and two lines broad, but a portion of the funis was compressed. An important distinction, however, is to be made. If there is a band of ecchymosis around the neck, it ought not to form a perfect circle, but the two ends should cross one another. If the circle is perfect, we must suspect that the impress is due to a murderous hand, and this suspicion will be strengthened, if the lungs are found distended with air, and containing blood, together with all the other signs of life having gone on after delivery. As to supposed death from the shortness of the umbilical cord, the physician cannot give an opinion on this point, if the cord is not submitted to his examination. In the cases the most favourable for forming an opinion, his decision must still be doubtful; for all that he knows concerning the relation of the funis to the axis of the pelvis, concerning the dimensions of the fœtus, the state of the funis itself, the probable insertion of the placenta in the uterus, and all that respects delivery, will not allow him to decide if the child has died from tension of the funis during labour, or from some other cause.

*Delivery by the Feet.* Although delivery by the feet is frequently fatal to the child, medical jurists have taken but little pains to ascertain the effects of this kind of death. It is true that respiration in the vagina can very rarely take place in such cases, yet its possibility cannot be altogether denied. In the case reported by Pyl, all the signs of this variety of delivery may be recognised, and the question appears to have been well answered. In the case quoted by Batner, the midwife had extracted the fœtus. In the report, the compression of the cord, and the probable tension of the spinal marrow, have been passed over. When no trace of violence can be discovered, we may conclude that the death of a new-born child arises from a footling delivery, if, in addition to the signs of death caused by compression of the funis, a swelling is observed in the genitals and scrotum of the male, or in the labia of a female. Additional information may also be acquired from the circumstances attending the delivery, and from an examination of the genital parts of the mother.—*Abridged from the Archives Générales.*

EFFICACY OF THE EXTRACT OF BELLADONNA IN STRANGULATED HERNIA.

Dr. Fränkel, of Elberfeld, has tried the external use of the extract of belladonna with success, in cases where the taxis was ineffectual.

Case I. A healthy countrywoman, æt. forty-two, was struck upon the right hypochondrium by the axle-tree of a cart, and thrown upon the ground. Her countenance was pale, the extremities cold, the pulse small and spasmodically contracted; and she vomited bitter matter, mixed with mucus. In the left groin there was an incarcerated hernia, produced by the accident, as the patient expressly declared that there was none before. Venesection, leeches, poultices, and aperient clysters, were employed, and a saturated solution of carbonate of potass internally administered, with the effect of relieving the pain in the right hypochondrium, but the hernia was still irreducible. Feculent vomiting and other bad symptoms came on, and Dr. Fränkel consequently proposed the operation; but as this was not consented to by the patient and her friends, he gave occasional doses of cherry-laurel water, and rubbed in belladonna ointment upon the hernial tumour.* The symptoms soon gave way, and the hernia was reduced. This case was encouraging, still it was but a single case, and it might be said that the hernia would have been reduced without the belladonna. Another opportunity for trying its efficacy soon occurred.

Case II. A strong woman, æt. thirty-six, the mother of six children, had a femoral hernia, but did not wear a truss, as the gut came down only during her pregnancies. One night, however, when getting out of bed suddenly, the hernia became incarcerated. The operation was thought necessary, but the patient would not submit to it, and Dr. Fränkel was called in. The belladonna was again administered, and was again successful, though the incarceration was of eight days' standing.

Case III. The patient was a female of about fifty, who had long suffered from a crural hernia of the right side. She had long been unable to wear a truss, from the induration of some inguinal glands in the vicinity of the hernia. Leeches were applied, and the ointment rubbed in; and on the fifth day the vomiting ceased, and evacuations by stool took place. The hernia was reduced;

* What the strength of the ointment was we do not know, for there is obviously a misprint in the original, which runs as follows: *welche aus einer* 3 *Unguentum altheae und zwei* ʒ *extr. belladonnae bereitet war.—Journal für Chirurgie, &c.* Band xx. p. 561.—[*Ed. Med. Quart. Rev.*]

some of the inguinal glands suppurated, but healed under the use of bran poultices.

Case iv. The patient was a very old, thin and feeble woman, suffering under umbilical hernia. Here too leeches, poultices, and the extr. bellad. succeeded in making the hernia reducible. Dr. Fränkel states that he has used this remedy in six cases, and always successfully, but narrates only the four of which we have given an abstract. He refers to four cases of strangulated hernia given by Fuget Duponget, in the *Revue Médicale* for November 1831, in which linen, soaked in a solution of extr. bellad. was successfully applied to the abdominal ring. Our author, however, prefers rubbing in the ointment.—*Graefe und Walther's Journal.* Band. xx. Heft. iv.

### ON THE DIVISION OF THE TENDO ACHILLIS AS A MEANS OF CURING CLUB-FOOT.

The division of tendons as a means of curing certain deformities has been rescued of late by Delpech from the oblivion into which it had fallen. He lays down the following rules for its performance: The tendon should not be exposed, nor should the section be made in a direction parallel to the wound of the skin, lest exfoliation should take place. Immediately after the division, the two portions should be placed in contact, and retained there until they are consolidated. This union is accomplished by the formation of a fibrous substance, of a soft consistence; and it is during this state that gradual extension must be made, until the deficiency of the length of the muscular fibre is repaired, and afterwards continued till the intermediate substance has become perfectly solid.

The method adopted by M. Delpech did not, however, fulfil all his indications; for, in the case of a boy whose tendo Achillis he divided, the operation was followed by sloughing and tedious suppuration. The boy being laid upon his stomach, the operator plunged a bistoury under the tendon, dividing the skin for about an inch on each side of it; he then withdrew the instrument, and reintroducing a convex-bladed knife with the cutting edge towards the tendon, divided it from within outwards. Here, though the skin over the tendon was not injured, the tendon exfoliated, the parts suppurated, and the cicatrix afterwards impeded the motion of the foot.

Dr. Stromeyer has modified this operation of Delpech in a very ingenious manner. The following are some of his successful cases.

Case i. George Ehlers, aged nineteen years, came under the doctor's care in October, 1830, with an affection of his left foot, which had existed since his fourth year, and resisted every local and constitutional treatment. The toes were bent inwards and downwards, and the foot itself was so turned that its external margin was brought into a line with the axis of the leg, and so extended by the contraction of the gastrocnemii muscles, that both its edges were in a straight line with the anterior surface of the leg.

Above the outer margin of the foot were two callosities, arising from the pressure of the patient's walking on that part at the onset of his disease. The joint was nearly immoveable, and the leg much wasted. There was some curvature of the knee, from the use of a wooden leg, to which he had had recourse for five years. The operation was performed on the 28th of February, 1831. The patient being seated on a table before the operator, an assistant held the knee firmly, while another endeavoured to flex the foot, so that the tendo Achillis was rendered very prominent. A pointed convex bistoury was inserted underneath the tendon, about two inches from its attachment, dividing it as it passed along. Care was taken to make no external wound on the opposite side, and to confine the other to the size of the blade of the bistoury. Very little blood was lost. The tendon retracted but slightly, scarcely three quarters of an inch, when the foot was flexed, and during its extension the parts readily came into contact. The wound was covered with plaster; a long compress was placed on each side of the tendon, with a figure of 8 bandage, which answered the two purposes of pressing on the part and keeping up extension of the foot.

On the third day the wound was healed; the tendons were slightly swollen, but as yet there was no union between the two portions, a fact manifested by the motion of the foot not being communicated to the upper portion.

On the sixth day the swelling had subsided, and there was a slight degree of union. On the tenth all tenderness had ceased, and the patient could readily move his foot.

At this period Dr. Stromeyer commenced his extension, by means of an apparatus hereafter to be described. The utmost caution was employed during its application, lest the recent adhesions between the surfaces of the tendon should be ruptured. At the end of a week, however, more force could be used without occasioning any severe pain, and in the course of eight weeks the foot was brought to a right angle with the leg. An iron boot was then applied, which could be made to act, with the power of a screw, in diminishing this angle, without destroying the mobility of the joint. With this boot the patient could walk across the room; but, of course, suffered at first from weakness and œdema of the limb, occasioned by its long inaction. This swelling subsided at the end of two months, but it was even then impossible to form any very accurate opinion as to the size of the tendon, or the length of its connecting substance. It might, however, be observed that the gastrocnemii muscles on that side were much higher than on the opposite leg; proving that this power of flexion of the ankle-joint did not depend upon any elongation of the muscular fibre, but of the tendon only.

The patient laid aside his boot at the end of six months, and at the period of the publication of his case (eighteen months after the operation,) he had a perfect use of the joint, the newly formed substance having shown no disposition to contract.

Case ii. M. B. Blumenthal, æt. thirty-two years, consulted Dr. Stromeyer, in May, 1832, for a similar affection of his left ankle. Its origin was attributed to certain convulsions, from which the patient suffered during infancy, but it had gradually augmented until it had confined him to his bed. The state of the foot was very similar to the former case: the heel never touched the ground, but the muscles were only slightly wasted, and the joint remained perfectly moveable. The operation was performed on the 12th of June, 1832, and the only difference in the mode of its accomplishment was, that the bistoury was inserted at a distance of three inches from the tendon, in order to remove the wounds from the pressure of the bandages. During the flexion of the foot the portions of the tendon were separated, but, on its extension, they were readily brought into contact. The parts were subsequently kept in a state of perfect rest by means of a splint applied to the anterior surface of the leg and foot.

During the night the patient experienced slight cramp in the leg, and on the second day the ends of the tendon were discovered not to be in contact. A bandage was applied round the whole limb, in order to prevent their muscular contractions, but was ineffectual. It was therefore removed, and they did not return under the use of the splint only.

By the fifth day union had commenced, and on the tenth extension was resorted to, and continued during five weeks. The boot was then applied as in the former case, and in ten weeks the foot was brought to a right angle with the leg, and the sole of the foot was planted horizontally on the ground in walking.

The apparatus employed for the extension by Dr. Stromeyer consists of a splint longer than the leg, and terminated inferiorly by a rounded notch for the external part of the leg and the prominence of the malleolus. A moveable wooden sole was situated between projections bordering this notch, which could form various angles with the perpendicular portion of the splint. To this sole a small windlass is attached, the cords of which pass over pullies fixed to the upper and anterior portion of the splint, so that, by turning the handle of the former, the sole may be brought to a right angle with the other part of the apparatus. An iron catch falls into the teeth of the wheel attached to the windlass, so as to prevent its returning on itself. By means of this (care being previously taken to pad and bandage the limb,) powerful extension may be employed upon the tendon, and graduated at pleasure.—*Abridged from the Archives Génerales.*

### CONVERSION OF THE RIGHT LUNG INTO AN ENCEPHALOID STRUCTURE.

John Keating, aged thirty-six, of a muscular form, a printer, admitted on the 1st of May, 1833, into the Meath Hospital, which he had left in the beginning of April, having then been in it nearly two months. He dates his illness from the summer of 1832, at which time he became subject to occasional pains in the right side of his chest, increased by deep inspiration. Last November he was attacked with cough, dyspnœa, hoarseness, slight expectoration, at first mucous, afterwards a little tinged with blood, and constipation of bowels. In a short time he observed also some œdema of the face and neck, rather greater on the right side, and on rising in the morning. This illness he attributed to over-exertion, want of rest, and cold caught by handling damp paper. The symptoms were a little relieved by venesection and a cough mixture. The attack however recurring, he came into the Meath Hospital in the month of February last, labouring under symptoms less urgent, but of the same character with those detailed below, on his second admission. He was treated by Dr. Stokes with venesection, moderate mercurialization, repeated blisters, &c., and went out slightly improved about the beginning of April. Being still however unable to work, and finding his symptoms returning with increased violence, he again came to hospital, and was placed under my care, the 1st May. His chief distress arose from excessive dyspnœa, almost amounting to orthopnœa; when he lay down the only position in which he could breathe tolerably was on the right side. After a few weeks he found it impossible even to do this; and for eighteen or twenty days before his death he sat in his bed night and day, leaning forward as far as possible, and supporting his head by means of a pillow placed on his knees. A state more piteous could scarcely be imagined. When admitted his dyspnœa was increased by the least exertion, which brought on palpitations of the heart. He had a dry cough, with occasional scanty expectoration slightly tinged with blood; no pain in chest, with the exception of slight stitches on making a full inspiration. He experienced some difficulty of swallowing, and referred the cause of obstruction to the lower part of the throat. There is no soreness in any part of the chest, but he complains of some pain about the right shoulder. His face is bloated, pale, and looks as if it were slightly œdematous; this, together with a certain appearance of the eyes, as if the balls were somewhat protruded from the sockets, and a marked dilatation of the nostrils during breathing, gives his countenance an expression of distress and suffering. The right jugular vein was much distended, as were the veins in the right axilla, but this symptom was chiefly remarkable on the surface of the belly, where two veins corresponding to the situation of the superior epigastric artery pursued a remarkably tortuous course along each side of the linea alba, being turgid, and dilated to the size of swans' quills.*

His bowels were constipated, and subject to griping pains.

* "This circumstance indicating some obstruction at the right side of the heart, I then considered as affording indubitable evidence of the disease of the heart itself. The dissection proved that the cause lay not in the heart, but in the impervious state of the right lung, in consequence of which, the black blood had its

Urine scanty and high coloured; loss of appetite; night sweats; slight thirst, tongue clean, pulse one hundred, regular, and compressible.

*Examination of Chest.* The intercostal spaces on the left side are more distinct, deeper, and more dilated in respiration, than those on the right; the latter, however, although not so well marked, are by no means obliterated, or distended by pressure from within. The right side of the chest measured about half an inch less than the left.

*Percussion.* Left side anteriorly, a clear sound everywhere, until we came within an inch of the sternal median line, where it became dull. Posteriorly, everywhere a clear sound. Right side, universally over every part, as dull as possible.

*Respiration.* Puerile over the whole of left side, except on approaching the sternal median line, where it assumes a tracheal character. This tracheal respiration is observed over a great part of the anterior part of the right side, where it is very loud and distinct above the mamma, feebler immediately below it, and is almost entirely lost still lower. On the posterior part of the right side, the loudness and tone of the respiration are not by any means so decidedly tracheal as anteriorly; to some, the sound heard appears to be more allied to bronchial respiration, and it is certainly bronchial in one part, near the spine. No rales are audible in any part of the chest.

*Voice.* At the upper and anterior part of the right side, the voice is resonant, approaching to, if not identical with bronchophony; elsewhere, nothing remarkable was observed with respect to the voice.

*Heart.* Pulsates in its natural situation, but its sounds are heard over a great extent, being audible under both clavicles, and over the whole of the right side. Right side of chest, during respiration, obviously moves much less than the left, and when he speaks, the hand placed on it feels the vibrations caused by the voice to be feebler on the right side than on the left.

The physical phenomena here detailed remained unvaried until his death, except that all traces of bronchial respiration soon disappeared from the right side of his chest, except at one spot near the spine, and where any thing was heard in other parts it was now evidently a tracheal wheezing which marked all other sounds.—*Dr. Graves, in Dublin Journal, No.* 12.

exit from the right side impeded; none, or nearly none, passing through the pulmonary artery to the right lung. In truth, engorgement of the venous system, although it may indicate an obstruction somewhere in the central portion of the system of black blood, yet it by no means points out the exact seat of that obstruction: the obstruction may occasionally be even on the left side of the heart. With regard to the serpentine course of the abdominal veins above described, I find several such cases recorded, particularly one by Dr. Wright, of Baltimore, in his contributions to cardiac pathology, and one of a very remarkable nature by M. Renaud, in which the superficial veins of the abdominal parietes carried on a collateral circulation where the vena cava was obliterated."

[The remainder of the case may be told in a few words. The patient died on the 15th of July, and on dissection the right lung was found to be converted into an encephaloid mass weighing more than six pounds. "The heart was pale, and rather atrophied." Besides the other symptoms commonly supposed to denote disease of the heart, a *bruit de soufflet* had been observed, chiefly at the right side of the heart. The diagnosis which had been formed were "aneurism, circumscribed pleuritic affection, and enlargement of the heart; pleuropneumonia, pleurisy, and hepatization, in consequence of previous pneumonia, solidification from tubercles, &c." It must be confessed, that if stethoscopy in general be in its infancy, the cardiac division of the science is in an embryo state; and the lovers of truth must at present propound their diagnosis founded on rasping, cooing, or bellows' sounds, with doubt and modesty.—Ed. *Med. Quart. Rev.*]

### CHRONIC DISORDERS OF THE HEART.

Chronic disorder of the heart, whether its action is increased, diminished, or oppressed, is produced by disorder of the stomach, of the liver, or of the bowels.

1. The stomach, being disordered in some individuals, the heart's action is suddenly oppressed or suspended.

I have known it instantly suspended by pork, by tongue, by rich pastry, by heavy dumpling, or any indigestible substance of that kind. In some of these cases the person dies as suddenly as if he were shot through the brain, or through the heart itself. One of the first cases of typhus fever I attended was in a schoolfellow and friend of mine. He was convalescent, and as I was ignorant of this connexion between disorder of the stomach and disorder of the heart, I neglected to give any particular directions as to the quality and quantity of his diet during the state of convalescence. I was sent for one day, and upon arrival I found that he was dead: he expired instantly after having eaten a large and indigestible meal. I have seen other cases of this kind. The only way to save the patient, if you chance to be present, is by diffusible stimulants, as a glass of brandy, the patient being placed in a recumbent posture.

In other cases great oppression of the heart only occurs. The patient is pale over the whole surface of the body; sometimes he falls down suddenly and faints, with a dilated pupil, a blanched conjunctiva, a weak respiration, and a small, struggling, irregular pulse. I saw a gentleman who had been travelling a long way, and at the end of his journey ate some veal pie with a heavy crust. He dropped down insensible, his breathing was feeble, his pulse struggling, his eye blanched, his pupil dilated, and he was in a slight degree convulsed. If you can act on the stomach by an emetic, it is the best plan; but if the stomach be not obedient to an emetic, the patient has the best chance from diffusible stimulants. When persons die in this state you generally find the brain more or less gorged with blood, or an effusion of thin serum into the ventricles or between the membranes of the brain.

2. More commonly than this, there is an intermittent or an inordinate pulse from disorder of the stomach. A person has a torpid liver, uneasiness about the stomach, and a torpid colon: he is subject to very great irregularity of the heart's action, to flushes of heat in the face, to coldness of the feet, and his pulse is frightfully intermittent after a meal. It beats once, twice, thrice; then there is a sudden stop, and then it goes on again. It is to be remedied by acting gently on the liver and bowels, and regulating the diet.

3. Sometimes the heart's action is what is called inordinate. One pulsation is strong, the next weak; then three or four pulsations are rapidly performed, and are succeeded by three or four slow pulsations. This is exceedingly common, from disorder of the stomach. So long ago as the year 1811, I saw a lady who for a long time had been subject to violent palpitations of the heart, and extreme irregularity of its action, so that at length she was unable to walk across the room without assistance. Every now and then she was suddenly seized with great difficulty of breathing, and coldness of the hands and feet, her face was flushed, she was perfectly powerless, and sunk from off her chair upon the ground. She went on in this way for twelve months; she had consulted several physicians, who agreed that she had organic disease of the heart. When I saw her, I thought it might possibly depend upon the stomach; and upon enquiring into her diet, I found it all originated from a small quantity of pastry which she always took once in twenty-four hours, or oftener. By withdrawing this, and giving her a small quantity of animal food, she recovered very rapidly indeed. I saw a lady in London with the same symptoms, with a torpid colon, and a foul tongue. She was supposed to labour under organic disease of the heart by the physicians whom she had consulted. I regulated the diet carefully; I acted gently on the liver, and moderately on the bowels; and all her symptoms disappeared with great rapidity.

If, then, you see a case in which there is irregularity of the heart's action, with a foul tongue, with irregularity of the liver, the stools showing either a deficiency or a depravity of bile; when, indeed, there are any indications of disorder of the stomach, liver, and bowels, be careful of giving an opinion as to the existence of organic disease of the heart. In all these cases, try first to remove the disorder of the stomach, liver, and bowels.

4. The heart's action is preternaturally increased from disorder of the stomach, liver, and bowels. Chronic inflammation, seated in different parts, is chiefly maintained by excitement of the mucous membrane of the stomach operating on the heart's action; and this excitement of the stomach is produced and maintained by daily errors of diet and drinks. A great many chronic inflammations arise, or, if not, are maintained thus; therefore, in all chronic inflammations, support the strength, without exciting the heart's action, by carefully regulating the diet. In all chronic diseases, that diet is prejudicial which increases the heart's action and the animal heat simultaneously.—*Armstrong's Lectures.*

### PATHOLOGY OF THE FŒTUS.

Dr. Ollivier, of Angers, has some observations on this subject in the *Archives Générales* for May, and narrates three cases of disease in the fœtus not derived from the mother.

CASE I. *Ulceration of the Skin.* On the 24th of April, 1828, a child was brought to Dr. Ollivier, labouring under a rare variety of club-foot, the back of each foot being directly applied to the anterior part of the leg. Above the left malleolus externus, and in the fold formed by the forced flexion of the foot upon the leg, there were two ulcerations of the skin, with a grey centre, and very red, bloody edges, looking like a fresh burn of the second degree. The right foot internally, and over the whole extent of its dorsal surface, presented a large scar of a yellowish grey, surrounded by a very red and bleeding inflammatory edge. This disease of the skin, like the one in the other leg, had the greatest resemblance to a recent burn.

Dr. Ollivier treated the case successfully by ordering the feet to be extended by a bandage, and the ulcerated surfaces to be covered with Goulard's cerate.

The author observes that this case shows one of the uses of the liquor amnii. Under some circumstances, the absence of the contact of atmospheric air makes the skin assume the characters of a mucous membrane. The skin becomes red, inflames, and softens; the epidermis is no longer visible, and its surface becomes covered with a copious exudation, quite similar to the mucus which labricates mucous membranes. This may be seen in the deep folds caused by the flexion of the limbs in very fat infants; and the same phenomenon is caused by muscular contractions of long standing: in fact, wherever the skin, by being in contact with itself, is withdrawn from the direct action of the air. The curious case just related shows that a faulty position of the limbs of the fœtus produces a similar effect, by withdrawing the skin from the influence of the fluid which moistens it, and performs the office of the atmosphere during the intra-uterine existence of the fœtus. This is, no doubt, the origin of those preternatural adhesions which accompany some monstrosities.

CASE II. *Warty Excrescences over a great part of the Body.* On the 12th of last April Dr. Ollivier was desired, by *M. le Procureur du Roi*, to open the body of a fœtus found in the Seine the day before, in order to ascertain the cause of its death. It seemed to have arrived at its full time, and to have been in the water about three weeks.

On the whole front of the chest, and on the abdomen, especially its upper part, there were an infinite number of warts of a greyish white, several of which were as large as a lentil. They all had pedicles, and the largest were fissured through the greater part of their substance. On detaching the epidermis, which putrefaction had separated, from the skin, it was seen that all these excrescences penetrated it, without being covered by it; and dissection showed that they were rooted in the skin itself, and even in the subcutaneous cellular tissue. But this warty vegetation was not found only on the anterior part of the chest and abdomen; it likewise existed on both shoulders, and both arms, as far as the elbow, particularly outwards and rather backwards; and on the thighs and nates, also outwards and backwards only. These regions were covered on the right and left for the same extent with a considerable number of small warts, which gave the skin a grained appearance; several of them had the yellowish colour of ephelides, so that at a distance it might have been thought that the skin was suffering merely from this discoloration. All the viscera of this fœtus were healthy.

If the affection of the skin just described resembles, in its anatomical characters, the disease which occurs at other periods of life, it is remarkably different in its development; for warts, which are so often found on the surface of the skin, ordinarily occur only on some isolated points, and do not cover, as they did in this case, a great portion of the body, occupying in a symmetrical manner the same region on the four limbs. The only case in an adult having some resemblance to this one is mentioned by Pechlin, (*Obs. Phys. Med.* Hamburg, 1641, 4to. Lib. 2. Obs. 44.) who saw a surgeon, the whole surface of whose body was covered with a great number of warty excrescences.

The case narrated above shows, in contradiction to the opinion of Corry, that warts may be congenital: *Nunquam connatæ sunt verrucæ,* he says, (*Tract. de Morb. Cut.* Paris, 1777, 4to. p. 542.) It is possible that cases of this kind may have been taken for instances of congenital venereal disease; but here no mistake could be made, for there was no resemblance between these and syphilitic warts.

Case III. *Subcutaneous Abscess in a Fœtus about Three and a half Months Old.* The author, in making a medico-legal examination of this fœtus, found on the right and anterior half of the neck a tumour as big as a hazel-nut, its yellowish white colour forming a strong contrast with the reddish tint of the rest of the body. The tumour was soft, with fluctuation, and extended from the base of the lower jaw to the right sterno-clavicular articulation. An incision was made, and gave exit to a white, creamy, inodorous pus, situated under the skin, which it separated from the subjacent muscles. The integuments around this collection of pus were in their natural state.

It may be suspected, indeed, that this abscess was caused by attempts to procure abortion; but it is just as probable that it may have been the consequence of a spontaneous phlegmon.

### PHLEGMASIA DOLENS IN MEN.

The Phlegmasia Dolens, that white elastic swelling, generally of one, very rarely of both the lower limbs, attended with great pain and soreness, is supposed to be peculiar to female nature, indeed to women after lying-in, and was formerly considered and miscalled a "Depôt du lait."

But a more accurate pathology has exploded the notion of its being a deposit of milk, and has assigned causes for the disease, which do not preclude the other sex from a liability to it. Indeed, a most respectable general practitioner has expressed such a belief, and I am much mistaken if I have not seen it in three instances, within the last few years, in men; and if the suggestions of some intelligent practical writers, who have attributed the disease to an inflammation of the veins of the pelvis be correct, there is no reason why men should not occasionally contract the malady, though it will be easily admitted that the long-continued pressure of the pregnant uterus on the iliac veins, and the violent change which that part of the female system undergoes by parturition, must render women more frequently the subjects of this complaint.

Perhaps you will allow me to give two of these cases in some detail; and I beg you to bear in mind the notion of an inflammation of the veins of the pelvis as the origin of the painful affection; because I think their history serves much to confirm the correctness and truth of this notion.

The late Earl of L. suffered with this disease many years before his death, and bore marks of it to the last, in a swelling of the left leg and thigh, and in the varicose state of the veins from the ankle to the groin. He was attended by the late Dr. Pemberton in the first instance, and the symptoms were palliated from time to time; but he remained subject to repeated attacks of the same painful malady; and I am persuaded that the obstruction to the circulation of the blood, occasioned by the original inflammation, gave rise at length to that disease of the brain which incapacitated him for the business of his great office, and ultimately deprived him of life.

When I first attended him, some three years before his death, I found him subject to temporary congestions in the liver, which were relieved by small repeated doses of calomel, followed by purgative draughts containing neutral salts. But there was something extraordinary in his pulse which attracted my particular attention. It was most unusually slow, beating only forty-four pulsations in a minute, whereas I learnt that the original habit of it was to give seventy-four strokes in that space of time. This was ingeniously conjectured by Sir Astley Cooper, who had attended him with Dr. Pemberton, and had witnessed repeated attacks of the inflammation of the veins, to be attributable to an obliteration of the external iliac vein of the side affected, by which the blood was returned to the heart more slowly, and the vital organ was not stimulated thereby to contract itself till after longer intervals than had been its custom. The good reason and propriety of this conjecture was abundantly confirmed by examination of Lord L.'s body after death, when the left external iliac vein was found to be impervious for several inches, and, what is remarkable, the corresponding vein on the right side was ossified.

It is not improbable that the stroke of apoplexy which brought his life into imminent hazard when it occurred, and which destroyed his mental powers for the whole year during which he survived it, was referable to the same obstruction to the return of the blood towards the heart from the lower extremities; nor was it unlikely that a large accumulation of blood in the sinuses of the brain (in consequence of an impediment to its free ingress from the vena cava descendens, into the right auricle, caused by the heart's preternatural delay in contracting,) should occasion an effusion of serum into the brain. This was the case in fact; and at least four ounces of lymph were deposited in its substance, in an unnatural cavity extending from the roof of the ventricle to the pia maternal covering of it. Lord L., sometime previously to the apoplectic seizure, had complained of an imperfection in his vision, and used to remark that he missed a word or two in every line; but, after the blow was struck, he lost the power of speech almost altogether. Epileptic fits followed, at uncertain intervals, and in one of these he expired. Alas! how fearfully and how wonderfully are we made! and on what a thread does this proud distinction of man, his reason, and his life, depend! What momentous consequences do sometimes follow the slightest derangement of the economy of our curious fabric! This inflammation of the vein, from whatever cause it arose, (the most probable one was, exposure to a cold March wind in a rather thinner dress than usual,) appeared to give way to appropriate remedies, and was not thought of any importance beyond the pain and inconvenience which it occasioned at the moment; but it was destined to produce a tragedy, sometime after, of unusual interest and distress. Lord L. married subsequently to the first attack of the disease, and was directing the affairs of this great nation at the height of its glory, when the matured consequences of this disturbance of the circulation, by a common cold, deprived him of his intellect and of his life.

Another case of this disease presented itself to me, in the person of an officer of high military reputation, who fell ill under symptoms of an inflammation of his chest. He had already been bled, and had taken physic, when I saw him, and was complaining of acute pain in the region of the liver. This was met by a further loss of blood by cupping the right side; soon after which a deep-seated pain attacked him in the left groin; here sixteen leeches were applied, and the part was fomented. On the following day, the thigh and leg were considerably swollen; some knots could be felt in the course of the veins, and the lymphatics of the surface manifested themselves in red streaks. Here again the inflammation and soreness were treated by more leeches, and cold lotions; and a necessity for their repetition occurred three several times more on account of the pain, once again above the knee, twice in the leg.

At length the fire was extinguished, but the limb has continued swoln, though to a less degree, ever since. However, some baths on the Continent, and a bandage, have reduced the inconvenience so much, that it interferes now but little with the comforts of life. But my patient returned to town, this last autumn, under considerable anxiety and alarm, on account of a notable intermission which had been discovered in his pulse; the importance or no importance of which some time will be necessary to ascertain; but this symptom will induce me to look with suspicion upon, and to watch with jealous care, any affection of the head, should it arise.

As to inflammation of the veins, generally, it is not my purpose to discuss this question on the present occasion, nor to enter into consideration of the various opinions which pathologists have entertained of the nature of the symptoms which arise in that state. We all know that injuries done to veins by accidents are apt to produce a disease which proceeds most rapidly to the destruction of life, and that this is much less frequently the case where the inflammation has arisen spontaneously. Probably the admission of air into their cavities, as would often be the result of an injury, may make the difference. No; my object, in this short paper, has been merely to assist in doing away the opinion that phlegmasia dolens is peculiar to women; and to confirm, as far as these two cases may be thought to confirm it, the later and better philosophy of the disorder.

I would add, that I think it worth your future attentive consideration, and inquiry, whether the irregular intermittent pulse, so frequently observed in the decline of life, may not be traceable to some past unheeded inflammation in an important vein, and to a consequent impermeability or obliteration of its channel.—*Sir H. Halford's Essays and Orations.*

### HYPERTROPHY OF THE MAMMÆ.

A case of this nature came under my observation in May last. The subject of the affection was a girl nineteen years old, a respectable farmer's daughter; she was of good constitution, rather robust make, and sanguine temperament. From the account given by the surgeon in attendance, it appeared that she had never menstruated, but had occasionally suffered from pains in the head, back, and abdomen, which, so far as he was enabled to judge during a period of three or four months, seemed to coincide with those which usually forerun and accompany the eruption of the menses, observing likewise a similar periodicity in their recurrence. The treatment he had adopted had been limited to emmenagogue pills of aloes, myrrh, and calomel, with saline aperients, and the occasional application of leeches behind the ears. The enlargement of the breasts had been first noticed when she was seventeen years of age, their development having commenced at fifteen. The girl described her feelings in the breasts up to about her eighteenth year as rather pleasant, experiencing hot, tingling sensations in them. After this time she felt nothing to attract her attention to them, unless the occasional feeling of a dull, heavy pain in the breasts, which was generally the worst at those times when she was afflicted with the headachs and pains in the back. When I visited her she was in perfect health; but her appearance was rendered remarkable by the extraordinary size of the mammæ, which she represented as exceedingly burthensome when deprived of the support afforded by her stays. On measuring the glands while she lay in bed, the circumference of the right breast was found to be upwards of twenty-three inches, and that of the left about twenty-three inches; while retained on the chest by the hand, their height amounted to seven and a half inches.

Conceiving that this was a case of postponed menstruation, in which the nutritive vessels of the mammæ had become vicarious of the excretory function of the uterus, and that their inordinate activity had now become the cause of the suspension of the catamenia, we determined on employing such remedies as might, by exciting the absorbents of the mammæ, counteract the undue textural deposition, at the same time that they would, by expediting the absorption of medicines given for the purpose of eliciting the uterine flux, the more speedily induce their operation. On the 18th May she was ordered to take ten grains of the ergot of rye twice, with one of the following pills thrice a day: ℞. Pilulæ Hydrargyri gr. xxiv., Iodini gr. xii. Morphiæ Muriatis, gr. j. Confect. Aromaticæ ℈ss. Ft. Massa in Pilulas xii. dividenda.

With these medicines she persevered until the 25th, when, having an attack of the symptoms of which she used to complain, I was requested to see her again, and then ordered her to be blooded to twelve ounces, and to be placed in the hip-bath, which produced very considerable relief, but no appearance of vaginal discharge. On the 28th, the pills were suspended, as her mouth had become affected by the mercury. She still continued the ergot until I saw her on the 3d June. At this time, more than a fortnight from the commencement of her treatment, there was no advancement made towards the removal of the cause of the affection; the breasts themselves seemed in no way altered; but, as she was still under the influence of mercurial fever, it was thought advisable to discontinue all medicinal treatment.

About the 16th of June she was directed to recommence the ergot of rye, taking ten grains thrice, instead of as previously, twice a day. With this she continued for a week, when, on experiencing a return of her periodical ailments, there was observed a slight appearance of sanguineous discharge from the vagina, which continued for four or five hours. She was now recommended to give up her medicines for a time, but to resume them every month for a few days before the succeeding menstrual evacuation was to be expected. With this advice she proceeded for two months, at each period having an imperfect vaginal discharge. On the third month she again began to take iodine, ten drops of the tincture three times a-day, in a wineglassful of water, and the ergot of rye as usual. Her menses appeared at the due time, and more abundant in quantity. From this time the ergot of rye was entirely discontinued. With the tincture of iodine she persevered for upwards of a month, when it was thought necessary, in consequence of some uneasiness of the throat and fauces, to suspend it. On examining the breasts, in September, they were found to have lost about one-sixth of their volume. As she appeared now to be doing well, and the natural operations of the organs to be established, she was advised to trust to her own physical energies for the completion of what seemed the commencement of a removal of her affection.

When I saw this female, in the latter end of November, she was in perfect health, her catamenia had continued to appear regularly, and her breasts had suffered a very surprising diminution; so much indeed, that she was not only relieved from the inconvenience of which she originally complained, but their volume was scarcely greater than the usual size.—*Monthly Archives.*

### TRIPLE QUOTIDIAN AGUE.

Charles Comins, æt. twenty-nine, an Irish labourer, applied to Dr. Duncan, on the 4th of March. He stated, that each day he had three distinct attacks of rigors, followed by heat and sweating; viz. at six A.M., eleven A.M., and six P.M. In July last he had worked at the harvest in the Black Fen; the first attack of ague took place three weeks ago; he had used no remedies.

He was ordered to take immediately a scruple of the Pulvis Purgans of the Dispensary Pharmacopœia (containing about sixteen grains of Jalap and four of Calomel,) and four grains of the Sulph. of Quinine every four hours.

On the 5th, he had only one attack of rigors, but on each of the three following days he had two attacks. At the end of a week the ague had entirely left him, but he continued the quinine, in diminished doses, for ten days longer. On the 5th of April the ague returned, but under the form of a double Quotidian only. It readily yielded to the quinine, given as before, after a brisk cathartic. —*The Liverpool Med. Journal, June.*

